# Personal and Clinical Determinants of Brace-Wearing Time in Adolescents with Idiopathic Scoliosis

**DOI:** 10.3390/s24010116

**Published:** 2023-12-25

**Authors:** Giulia Fregna, Sara Rossi Raccagni, Alessandra Negrini, Fabio Zaina, Stefano Negrini

**Affiliations:** 1 ISICO (Italian Scientific Spine Institute), 20141 Milan, Italy; sara.rossiraccagni@isico.it (S.R.R.); alessandra.negrini@isico.it (A.N.); fabio.zaina@isico.it (F.Z.); 2 Doctoral Program in Translational Neurosciences and Neurotechnologies, University of Ferrara, 44121 Ferrara, Italy; 3 Department of Biomedical, Surgical and Dental Sciences, University “La Statale”, 20122 Milan, Italy; stefano.negrini@unimi.it; 4 IRCCS Istituto Ortopedico Galeazzi, 20157 Milan, Italy

**Keywords:** idiopathic scoliosis, adolescence, brace, adherence, compliance, sensors

## Abstract

Adolescent idiopathic scoliosis (AIS) is a three-dimensional spine and trunk deformity. Bracing is an effective treatment for medium-degree curves. Thermal sensors help monitor patients’ adherence (compliance), a critical issue in bracing treatment. Some studies investigated adherence determinants but rarely through sensors or in highly adherent cohorts. We aimed to verify the influence of personal and clinical variables routinely registered by physicians on adherence to brace treatment in a large cohort of consecutive AIS patients from a highly adherent cohort. We performed a cross-sectional study of patients consecutively recruited in the last three years at a tertiary referral institute and treated with braces for one year. To ensure high adherence, for years, we have provided specific support to brace treatment through a series of cognitive-behavioural interventions for patients and parents. We used iButton thermal sensor systematic data collection to precisely analyse the real brace-wearing time. We included 514 adolescents, age 13.8 ± 1.6, with the worst scoliosis curve of 34.5 ± 10.3° Cobb. We found a 95% (95CI 60–101%) adherence to the brace prescription of 21.9 ± 1.7 h per day. Determinants included gender (91% vs. 84%; females vs. males) and age < 14 years (92% vs. 88%). Brace hours prescription, BMI, and all clinical variables (worst curve Cobb degrees, angle of trunk rotation, and TRACE index for aesthetics) did not influence adherence.

## 1. Introduction

Idiopathic scoliosis is a complex spinal and trunk deformity [1]. The adolescent type (AIS) impacts approximately 2% to 3% of individuals in the general population from age ten until they reach skeletal maturity [2]. When left untreated, AIS frequently progresses [3,4,5]. Scoliosis progressing above certain thresholds leads to severe trunk deformities, chronic back pain, and a decreased overall quality of life [1]. Moreover, scoliosis has an aesthetic impact [6] that can, in the long term, cause mental health issues [7,8,9]. The primary goal of conservative treatment for AIS is to halt or limit the progression of the spinal curvature, thereby improving the aesthetic appearance of the spine and reducing the risk of future back pain and disability in adulthood [1]. Moreover, conservative treatment aims to avoid surgery (spinal fusion) that straightens the spine in the frontal plane and restores the sagittal and horizontal planes. Nevertheless, surgery has possible immediate side effects [10,11] and restricts spinal movement function with potential long-term consequences [12].

The conservative treatment approach typically includes a step-by-step procedure, involving observation as the first approach in very low-degree curves, physiotherapeutic scoliosis-specific exercises (PSSEs) in low- to medium-degree curves, and the use of braces in medium- to high-degree curves [13]. Bracing is typically combined with PSSE to reduce the side effects of long-term immobilisation and improve the final results [14,15]. Numerous studies have demonstrated the efficacy of bracing in altering the natural progression of AIS and reducing the likelihood of surgical intervention. The extent of success with bracing, however, varies between studies, with significant differences in outcomes [1,3,16]. In the context of bracing, adherence is a crucial factor that plays a pivotal role in treatment success: the higher the adherence, the better the results [3,17,18,19,20]. We prefer the use of the term “adherence” as opposed to the classically used “compliance”, because it implies a more active role on the part of patients, which is especially relevant when considering bracing. Accurate monitoring of the number of hours a patient wears the brace is essential for assessing real adherence. In recent years, technological advancements have led to the development of electronic devices, such as thermal or pressure sensors, to provide more precise monitoring of treatment adherence, surpassing the limitations of traditional questionnaires or verbal reports.

Studies have shown that the use of temperature sensors can significantly improve adherence when patients are aware of the sensor’s presence, which becomes a motivational tool [21]. Moreover, patients receiving feedback and counselling based on their monitored adherence increase the devices’ use [22]. Another study showed that patients and their parents generally recognise the advantages of sensor-based monitoring and find it beneficial, with no adverse impact on the patient–physician relationship [23]. Research has also shown the importance of sensors in reducing the overestimation of adherence levels by healthcare providers, patients, and parents, which is common when sensor monitoring is not used [24]. A systematic review and meta-analysis conducted in 2022 confirmed that sensor monitoring stands out as the most promising approach among adherence-enhancing interventions, outperforming collaborative medical care, psychosocial interventions, and auto-adjusted braces [25]. Nevertheless, another systematic review focusing only on the subset of studies using sensor monitoring revealed significant variability in treatment adherence across different studies, only partially attributable to the currently known adherence-enhancing procedures [26].

Emerging evidence suggests that psychosocial factors at the outset of treatment may also influence adherence to treatments [27,28,29]. When it comes to bracing, factors such as high self-esteem, positive peer relationships, and negative attitudes towards the brace, especially by parents, have been linked to lower brace adherence [30]. In contrast, feelings of loneliness and heightened parental religiousness appear to be associated with improved brace wear [31]. Other characteristics like body image, socioeconomic status, family dynamics, and school performance did not exhibit significant associations with brace adherence [31].

Existing studies have widely reported varying levels of brace adherence [5,6,7,13,16,17,18,19]. To the best of our knowledge, the level of commitment to bracing in the studied population until now has not been considered as a factor that could change the determinants of adherence. These determinants could differ among populations independently of the effective supportive interventions. We hypothesised that a highly compliant population could show different contributing factors to a low compliant population.

This study aims to investigate the impact of personal and clinical variables, routinely documented by physicians during patient consultations, on brace adherence within a large cohort of consecutive AIS patients originating from a highly compliant population [32,33]. By examining these variables in-depth, we seek to enhance our understanding of the multifaceted aspects that shape adherence to brace treatment in AIS patients in order to improve brace management and tailor conservative therapy in a more effective way.

## 2. Materials and Methods

We performed a cross-sectional study on a cohort’s adherence to brace wear, involving consecutive participants who were treated with braces. The setting was a tertiary referral outpatient institute specialising in the conservative treatment of spinal disorders in Italy. The local Ethics Committee approved this study (code 466_2021) on 29 April 2021. We obtained written informed consent from all participants.

### 2.1. Participants

We searched our clinical charts to identify all consecutive participants who had their first consultation in the three years between November 2019 and November 2022, while adhering to the inclusion criteria. We considered participants eligible for study enrolment if they: (1) had AIS; (2) had been treated with a brace according to the Society on Scoliosis Orthopaedic and Rehabilitation Treatment (SOSORT) Guidelines [1]; (3) had a systematic quantitative recording of brace hours usage through a thermal sensor application; (4) were aged between 10 and 18 years at the start of treatment. We did not impose inclusion restrictions based on any other clinical criteria, including curve degrees, scoliosis pattern, or bone age.

We excluded subjects with: (1) secondary scoliosis; (2) neuromotor disorders; (3) missing radiographic or clinical data on the investigated variables; (4) participants with ≥2 weeks of missing sensor data, who declared they had removed the sensor to swim with the brace on, or due to serious health issues. The sensor is embedded in the brace, but it can be removed mostly to bathe or swim while wearing it during the summer holidays. Moreover, patients can have serious health issues (e.g., surgery, fractures) that necessitate the removal of the brace; Finally, we also excluded participants who started bracing during the summer because their doctors asked most of them to wear the brace only at night until September when they had to start the prescribed hours’ regimen. The individual charts did not report this oral indication, and it is consequently impossible to correctly judge treatment adherence.

### 2.2. Interventions

We used different braces as prescribed according to the SOSORT Guidelines. We followed an already described step-by-step strategy to define treatment intensity based on the risk factors identified during the clinical evaluation and shared decision-making with patients and families [1]. Curve magnitude and bone age are critical prognostic elements contributing, with curve location, to identifying the brace model and dosage needed. According to these variables, the SOSORT Guidelines provide different therapeutic options, ranging from the least aggressive approach (i.e., scoliosis-specific exercises, soft braces) to the most invasive one (part-time and full-time rigid braces). Within this therapeutic range, clinicians can modulate choices considering other fundamental variables such as the patient’s aesthetic impact, angle trunk rotation, and scoliosis stiffness.

We used the push-up corrective principle [34] obtained through the Sibilla and Sforzesco thoraco-lumbosacral orthosis. The distinction between the two braces comes only from the material and construction, making the first a rigid brace and the second a very rigid one. In the case of flexible curves up to 35 ± 5° Cobb degrees, we proposed the less invasive Sibilla. If curves were shown to be above this threshold, we prescribed the more demanding and effective Sforzesco brace [35]. For single lumbar/thoracolumbar curves within the range of the Sibilla brace, we alternatively used the derotational principle [34] implemented through the lumbosacral orthosis Progressive Action Short Brace (PASB). The brace dosage (prescription) is above 18 h per day (usually 20 to 24) at the first clinical consultation according to the curve’s magnitude: the higher the curve degree, the higher the number of prescribed hours per day. The dosage is subsequently reduced by a maximum of 2 h at every clinical follow-up (usually every six months), according to the results obtained. The brace is never reduced below 18 h per day until the patient reaches the Risser 3 stage with a maximum growth of 1 cm every six months. All patients are also prescribed PSSEs following the SEAS School [36].

We provide specific support for brace adherence through a series of cognitive-behavioural interventions to patients and parents as follows: (1) at brace prescription, they receive 30 to 45 min of counselling by an expert physiotherapist; (2) at brace delivery, there are another 10 to 15 min of counselling by the physician performing the clinical check of the brace (focusing on the technical efficacy, but also explicitly “to make the brace as less visible and cumbersome as possible”); (3) at each physiotherapy session (usually scheduled every 30 days), patients and parents receive specific counselling according to their needs, and time is dedicated to problem-solving related to everyday brace use; (4) at each professional encounter (by physicians, orthopaedic technicians, and physiotherapists) time and questions are devoted to accessing patients’ moods and performing brace problem-solving; the treating team provides reinforcement by showing photos taken at different treatment stages; moreover, adolescents are actively made aware of the importance of treatment for their future; (5) throughout all treatment, the team provides video and written support using various media, including personal emails, a specifically designed website, a blog, and a Facebook group; phone and email expert support are provided upon request according to individual needs; (6) at each medical consultation (usually every six months), physicians provide a progressive reduction in brace wearing as a reward system: the aim is for patients to exit the room smiling—when a decrease in hours is not expected to happen at the next visit for any reason, physicians already pre-alert patients to avoid disillusion; physicians also systematically explain to adolescents and their parents of the current situation and expectations and actively seek shared decision-making to involve everybody in the treatment choices and results, as far as possible.

### 2.3. Outcome Measure

The outcome was the actual adherence to treatment, as a percentage of the hours per day regimen prescribed by the treating physician. We calculated the actual adherence using a thermal sensor. Since 2010, we have introduced the iButton [32] into our clinical practice to precisely analyse the actual brace-wearing time for better tailoring dosage and treatment intensity. The iButton (Maxim Integrated Products, Inc.; 120 San Gabriel Drive, Sunnyvale, CA 94086, USA) is a commercially available small heat sensor (also composed of a battery and memory) installed in the brace under a pad that provides information on orthosis use according to the temperature data recorded.

We analysed the first year of therapy, which is the period with the greatest brace usage. We used published software [32] to analyse the iButton information.

### 2.4. Statistical Analysis

We verified each variable’s distribution and described the results using the average and standard deviation in case of a normal distribution, otherwise with median and 95% confidence intervals. We considered the effect of the variables collected at the first clinical consultation. Previous studies [33] have detected the assessed outcome (adherence) distribution in our population. Consequently, we decided to categorise the variables as follows:

- Personal variables:
  o The age in years: three groups: <14, 14–15, <15;
  o The bone age as judged according to the Risser stage: three groups: 0, 1–2, >2;
  o The Body Mass Index (BMI): three groups, according to the World Health Organization [37]: normal, overweight, underweight.

- Clinical variables:
  o The worst curve measured in degrees, according to Cobb [1]: three groups: <30°, 30–44°, >44°;
  o The Trunk Aesthetic Clinical Evaluation (TRACE) index, using the recently developed Rasch compatible version [38]: three groups: <45%; 45–55%, >55%;
  o The prominence measured in degrees according to Bunnell: three groups: <8°, 8–11°, >11°.

- Brace prescription:
  o The recorded hours of brace wearing: three groups: <19, 19–22, >22 h per day [1].

We finally performed a *t*-test for gender and a one-way ANOVA analysis for all the other categorical variables. We set a significance level of *p* < 0.05. For all analysis we used Stata Software ver. 14.0.

## 3. Results

**Figure 1:**
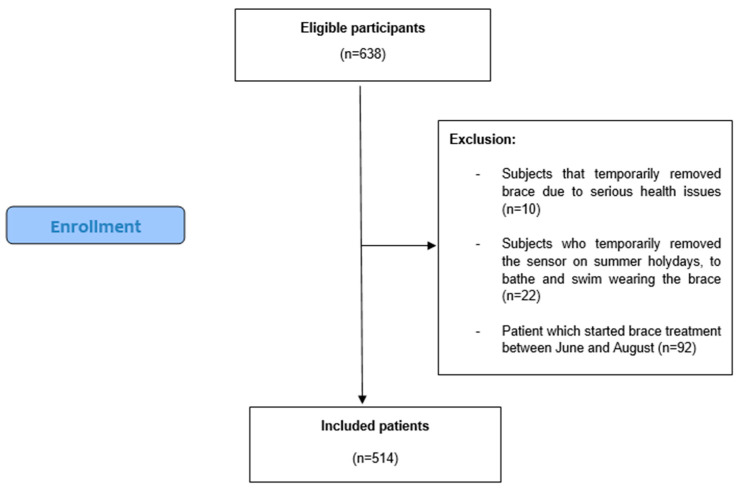
Flow chart of participant selection.

Our research involved a sample that initially comprised 638 eligible participants. These individuals were carefully selected based on our inclusion criteria, which were designed to ensure the relevance and reliability of this study’s results. As part of our data selection process, we excluded 92 participants who had commenced their treatment during the period between June and August. Additionally, 10 participants were excluded because they temporarily removed their braces, while 22 were excluded for removing the sensor. After these exclusions, our study ultimately focused on a cohort of 514 adolescents. The selection process is presented in Figure 1, while sample demographic and clinical characteristics are reported in Table 1.

Regarding the treatment process, our study revealed that participants were prescribed an average of 21.9 h of brace usage per day during the initial year of treatment. The measured median adherence rate was 95%, with a 95% confidence interval that spanned from 60% to 101% (values over 100% indicate patients who wore a brace for more than the hours prescribed). This translated into a real-world brace usage of 19.8 h per day. A total of 71% of the participants maintained an adherence rate of at least 90%, and 86% of the participants adhered to their treatment plan at a level of 80% or higher.

**Figure 2:**
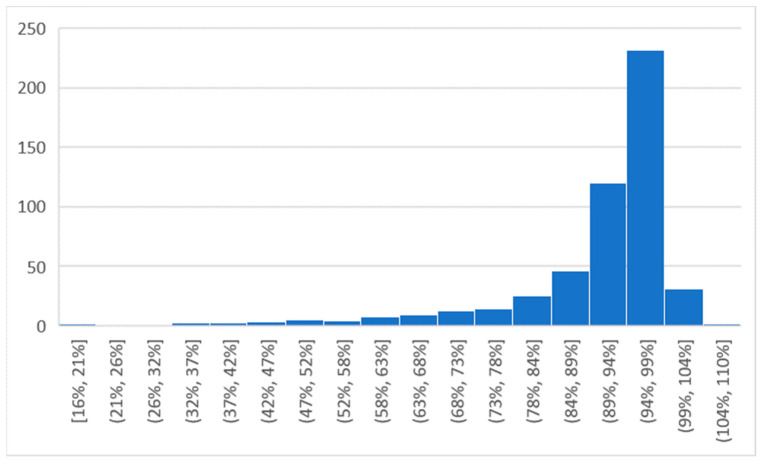
Distribution of adherence within the studied population. The number of patients per adherence rate classes, measured as a percentage between the brace hours reported in the medical prescription and the mean brace-wearing hours recorded by iButton, is reported.

Our findings regarding the primary outcome, adherence, are visually depicted in Figure 2. This figure illustrates the distribution of adherence levels among this study’s participants and highlights the variability in compliance within the cohort.

In our study, we observed variations in adherence between the two genders. Specifically, females exhibited a higher level, with a 91% adherence rate, compared to males, who demonstrated an adherence rate of 84%. This difference was statistically significant, with a *p*-value of less than 0.0001. We conducted a one-way analysis of variance (ANOVA), as presented in Table 2. This analysis revealed statistically significant differences among the various age categories and bone age measurements. The results indicated that younger participants tended to exhibit higher levels of adherence. Additionally, our analysis indicated a tendency toward statistically significant differences (with a *p*-value of less than 0.1) when considering the hours of brace prescription. The group with 19–22 h of prescription tended to have better adherence than the groups <19 h and >22 h. This suggests that the hours of brace prescription may play a role in influencing patient adherence, albeit not as pronounced as age and gender. Furthermore, our research did not identify significant differences in adherence related to BMI or other clinical variables, such as the severity of the scoliosis curve (Cobb degrees), prominence, or aesthetic impact as measured by the TRACE index.

## 4. Discussion

In our extensive investigation, we thoroughly examined the factors influencing adherence to bracing in a substantial cohort of 514 adolescents diagnosed with adolescent idiopathic scoliosis (AIS), all displaying high levels of adherence to brace wear. This study occurred in a specialized tertiary referral environment, where bracing treatment protocols employ cognitive-behavioural interventions to augment compliance. Our findings unveiled two crucial determinants: gender and age (whether chronological or bone age). We also found another potentially interesting variable (bracing hours prescription) warranting further study. Other variables, such as BMI and various clinical measures, failed to demonstrate statistical significance.

### 4.1. Gender Disparities

One of the central findings of our study pertains to the significant role of gender in determining adherence to bracing among AIS adolescents. Notably, females exhibited statistically and clinically significant higher adherence rates than their male counterparts, with a 7% advantage. This finding may be seen as somewhat surprising, especially when we consider earlier investigations on smaller cohorts, which did not uncover substantial gender-based discrepancies in adherence [15,17]. However, another study focusing on the daily adherence patterns of AIS patients found that males were more prone to inconsistent compliance than their female counterparts [19].

The divergence in these findings underscores the complexity of adherence behaviour among adolescents, particularly in the context of AIS treatment [39,40]. Factors such as psychological and social influences, individual attitudes toward medical treatment, and the perceived burden of bracing may contribute to these gender-based differences [41,42]. Further research into the underlying mechanisms behind these disparities is warranted, as it could inform the development of tailored interventions to enhance adherence among male AIS patients.

### 4.2. Age and Bone Age

Our study also highlights the significance of age, considered alongside bone age, as a determinant of bracing adherence among AIS adolescents. We observed that younger participants (those under 14 years of age) displayed higher adherence levels than their older counterparts, with a notable 4% difference in favour of the younger cohort. This finding adds another layer of complexity to the understanding of AIS bracing adherence, as previous research has yielded mixed results in this regard.

Some earlier studies, particularly those involving the use of temperature sensors on smaller samples, have reported a negative correlation between age and adherence. For example, a 2004 study involving 61 adolescents with AIS discovered a notable negative association between age and adherence [6]. In contrast, a recent investigation focused on the psychosocial determinants of adherence among 41 adolescents found no significant correlation between age and adherence [15].

The variability in these findings underscores the need for further research to elucidate the role of age in AIS bracing adherence. The obvious changes in adolescent psychological attitudes can play a significant role. It is also possible that individual differences in coping mechanisms, motivation, and social support networks may influence how age affects adherence behaviour [43]. Identifying the factors mediating the relationship between age and adherence could provide valuable insights for developing targeted interventions.

### 4.3. Bracing Hours Prescription

Another potentially interesting variable that emerged from our study is the “bracing hours prescription”. While our analysis did not yield statistically significant results for this parameter, there was a trend (*p* < 0.1) that warrants further investigation. The inconsistency in the existing literature regarding the impact of prescribed bracing hours on adherence underscores the complexity of this aspect of AIS treatment. Some studies have suggested that a more rigorous prescription involving longer daily bracing hours may result in improved adherence. However, others have failed to establish a clear relationship between the number of prescribed hours and actual adherence rates [17,18].

Our study, situated within the context of a tertiary referral setting and looking at a highly compliant population, did not conclusively demonstrate the significance of the “bracing hours prescription”. However, this variable should continue to be scrutinized in future research endeavours. Refining our understanding of the optimal balance between prescribed hours and real-world adherence is critical for optimizing AIS treatment outcomes.

### 4.4. The Broader Landscape of Adherence

Beyond our specific findings, it is essential to contextualize our study within the broader landscape of AIS bracing adherence. A recent systematic review [26], which included studies utilizing sensor-based monitoring for AIS patients, highlighted the substantial variation in adherence rates across different studies. These rates ranged from as low as 21.8% to as high as 93.9%, indicating a considerable disparity in adherence behaviours among AIS adolescents. Additionally, the review revealed a wide spectrum of actual brace-wearing durations, spanning from as low as 5.7 h per day to as high as 21 h per day. The variation in adherence rates and brace-wearing durations underscores the challenges associated with achieving consistent compliance with bracing protocols among AIS patients. Furthermore, it highlights the need for tailored interventions that address individual patients’ unique needs and barriers.

However, despite the considerable variation in adherence rates, the systematic review offered some potential solutions described in the literature [26]. Interventions such as counselling, education, and targeted exercises designed to enhance adherence have demonstrated effectiveness in improving both compliance rates (from 58.5% to 66%) and daily brace-wearing hours (from 11.9 to 15.1 h). This suggests that proactive efforts to support and educate AIS adolescents can yield positive results and ultimately contribute to better treatment outcomes.

### 4.5. Strengths and Limitations

Our study, like all research endeavours, is not without its limitations. The need to categorize variables due to the skewed outcome distribution reduced our analysis’s statistical power. The exclusion of subjects with ≥2 weeks of missing data may have partially influenced our findings; however, we excluded only the patients who declared they removed the sensor to bathe or interrupted brace therapy for serious health conditions. Patients with ≥2 weeks of missing data for other reasons (i.e., the choice to stop the therapy for a certain period for other reasons, like having more freedom) were not excluded. Consequently, the reliability and generalisability of the results should not be affected.

Additionally, our study relied on information available in clinical charts, which may not encompass all potential determinants of adherence. Nevertheless, the strengths of our study, including the involvement of a substantial number of consecutive participants and the examination of a specific population characterized by remarkably high adherence, provide valuable insights into the intricate landscape of AIS bracing adherence.

### 4.6. Implications for Future Research and Clinical Practice

In conclusion, our study has delved into the intricate web of factors influencing adherence to bracing among AIS adolescents. We have identified gender, age (considered alongside bone age), and the “bracing hours prescription” as critical determinants of adherence behaviour. These findings underscore the importance of tailoring interventions to address the specific needs of different patient populations. Looking ahead, it is imperative that future research builds upon these insights and seeks to unravel the underlying mechanisms that drive adherence behaviour among AIS patients. Furthermore, investigations should specifically focus on male patients and those above the age of 14 to explore whether alternative strategies can be developed to enhance their adherence further.

In the wider picture of AIS treatment, adherence to bracing protocols remains a critical piece of the puzzle. Achieving consistent and optimal adherence is a complex endeavour involving many factors beyond gender, age, and the specifics of the prescribed bracing regimen. As we continue to expand our understanding of these factors, we move one step closer to improving the quality of care and treatment outcomes for AIS adolescents around the world.

## Figures and Tables

**Table 1:** Demographic and clinical characteristics of the subjects included. BMI = Body Mass Index; TRACE = Trunk Aesthetic Clinical Evaluation; ATR = Angle Trunk Rotation; SD = standard deviation; CI = 95% confidence interval.

Variables	Total (N = 514)
Sex	Males (17.5%) Females (82.5%)
Age, years (mean, SD)	13.8 ± 1.6
Risser stage (p50, CI)	2 [0–4]
BMI, kg/sqm (mean, SD)	19.2 ± 3.2
Curve magnitude, Cobb degrees (mean, SD)	34.5° ± 10.3
TRACE index (mean, SD)	51.4% ± 14.6
ATR, Bunnell degrees (mean, SD)	10.1° ± 4.0

**Table 2:** One-way ANOVA results. We report adherence for variables with statistically significant differences between at least two categories. Degrees of freedom for all variables: 2–511. ° = degrees, NS = Not Significant.

Variable			Category Limits (Number): Adherence			F-Test	*p* Value
			Category 1	Category 2	Category 3		
Clinical	Worst curve	°Cobb	-			1.070	NS
	Prominence	°Bunnell	-			1.322	NS
	TRACE	Percent	-			0.741	NS
	Brace prescription	Hours per day	23–24 (49): 87%	20–22 (145): 92%	18–19 (320): 90%	2.538	<0.1
Personal	Age	Years	10–13 (291): 92%	14–15 (106): 88%	16–18 (117): 88%	5.434	<0.05
	Bone age	Risser	0 (166): 92%	1–2 (164): 90%	>2 (184): 89%	3.549	<0.05
	BMI	kg/cmq	-			2.171	NS

## Data Availability

We deposited the data presented in this study in Zenodo and they are openly available at https://zenodo.org/badge/DOI/10.5281/zenodo.8305775.svg.
